# Cysteine rich intestinal protein 2 links copper homeostasis to translational regulation in primary myoblasts

**DOI:** 10.17912/micropub.biology.001889

**Published:** 2025-11-19

**Authors:** Odette Verdejo-Torres, Teresita Padilla-Benavides

**Affiliations:** 1 Molecular Biology and Biochemistry, Wesleyan University, Middletown, Connecticut, United States

## Abstract

Copper (Cu) is an essential trace element for cellular metabolism, yet its roles in development are not fully defined. We identified murine cysteine-rich intestinal protein 2 (mCrip2) as a novel Cu-binding protein required for myoblast differentiation. RNA-seq of *mCrip2* -deficient cells revealed downregulation of ribosome biogenesis and translation genes. Loss of *mCrip2* reduced global protein synthesis by 20-30%, partially mimicking cycloheximide treatment. Interestingly, Cu supplementation restored protein synthesis despite persistent differentiation defects. These findings establish mCrip2 as a Cu-responsive regulator linking metal homeostasis to protein synthesis, suggesting a previously unrecognized connection between Cu availability and translational control in mammalian cells.

**Figure 1. mCrip2 -deficient differentiating myoblasts exhibit impaired protein synthesis, which is restored by Cu supplementation.**
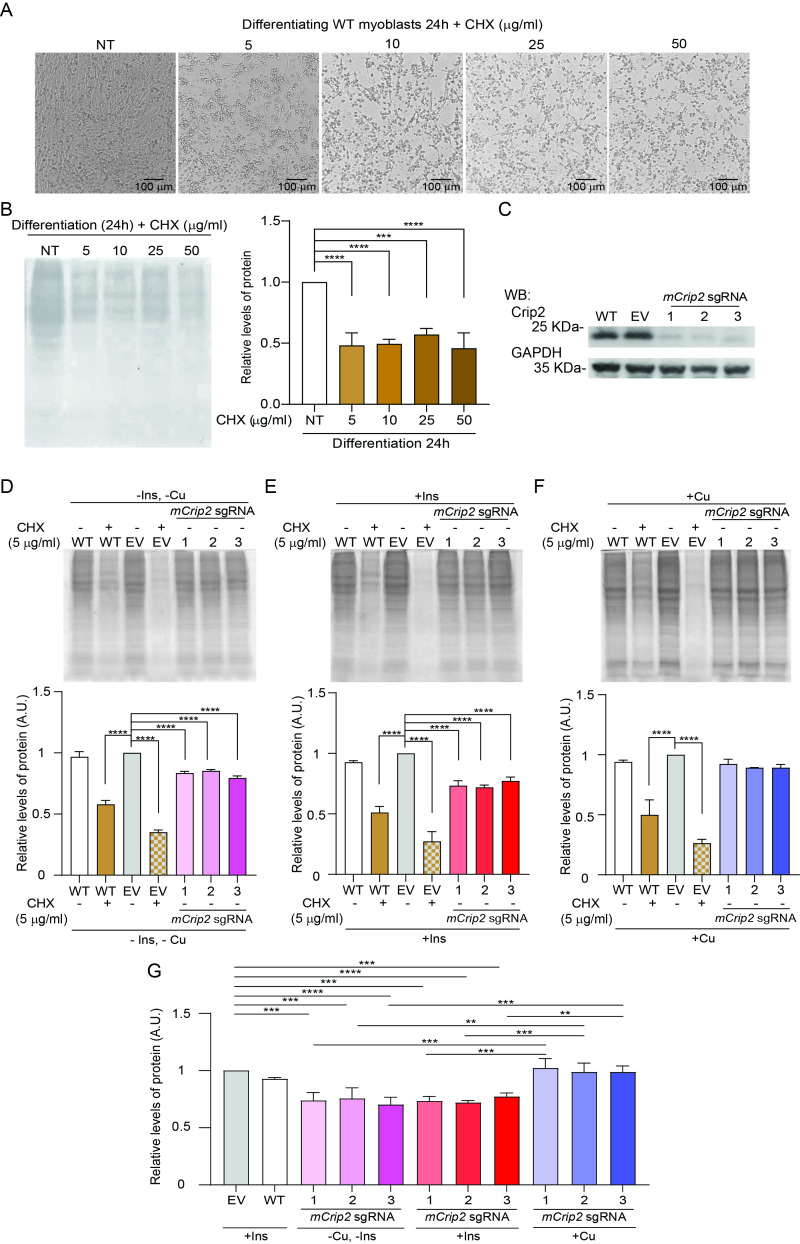
**(A)** Representative light microscopy images of wild-type (WT) myoblasts differentiated in the presence of increasing concentrations of cycloheximide (CHX). **(B)** Representative Coomassie Brilliant Blue-stained gel (left) and quantification (right) of total protein extracts from WT myoblasts differentiated under increasing CHX concentrations. **(C)** Representative western blot of mCrip2 protein levels in WT differentiating myoblasts and in cells transduced with empty vector (EV) or three independent sgRNAs targeting *mCrip2* . **(D-F)** Representative Coomassie Brilliant Blue gels (top) and corresponding quantification (bottom) showing total protein content in differentiating myoblasts under **(D)** basal conditions (no insulin, no Cu), **(E)** insulin supplementation, and **(F)** Cu supplementation. In all cases for Coomasie Brilliant Blue gels, the lysates were normalized by both total cell number and extraction volume (not by protein concentration) to assess total protein content per cell under each condition. WT and EV controls were treated with CHX (5 µg/ml), and comparisons were made between EV controls and *mCrip2* KO myoblasts in each condition. **(G)** Additional statistical analyses confirmed that total protein content in Cu-treated *mCrip2* -deficient cells was significantly higher than in untreated mCrip2-deficient cells. Overall, the data shows that Cu treatment restored total protein levels to near control values. Data represents N = 3 independent experiments. **P < 0.01, ***P < 0.001, ****P < 0.0001.

## Description

Copper (Cu) is a trace element essential for numerous biological processes mediated by Cu-dependent proteins (Festa & Thiele, 2011; Fraústo da Silva & Williams, 2001). Classic examples include cytochrome *c* oxidase (COX) for aerobic respiration, lysyl oxidases (LOXs) for collagen and elastin maturation, tyrosinase in melanin formation, as well as several Cu-binding proteins (CuBPs) in autophagy and transcriptional regulation (Carulli, 2025; Chen et al., 2021; Csiszar, 2001; Itoh et al., 2008; Leary et al., 2004; McCann, 2022; Morgada et al., 2015; Moriya et al., 2008; Ridge et al., 2008; Tavera-Montanez et al., 2019; Verdejo-Torres et al., 2024; Yuan et al., 2017)). Cu also contributes to catecholamine synthesis, peptide hormone modification, and redox balance through superoxide dismutases (SOD1/3) and ceruloplasmin (CP) (Fisher et al., 2018; Hatori et al., 2016; Orena et al., 1986; Ramos et al., 2016; Schmidt et al., 2018; Shiva et al., 2006; Vendelboe et al., 2016). Beyond these canonical roles, emerging evidence shows that Cu is required for tissue development, including muscle, intestine, and brain (Barnes et al., 2009; Barnes et al., 2005; Carulli, 2025; Hatori et al., 2016; Pierson et al., 2017; Schmidt et al., 2018; Tavera-Montanez et al., 2019; Verdejo-Torres et al., 2024; Vest et al., 2018; Whitlow et al., 2023). Maintaining appropriate Cu levels is critical, as deficiency impairs development while overload drives oxidative stress via Haber–Weiss and Fenton-like reactions, damaging [Fe–S] cluster proteins and DNA (Burton & Jauniaux, 2011; Gunther et al., 1995). To preserve homeostasis and support differentiation, cells depend on a network of transcriptional regulators, chaperones, cuproenzymes, and transporters (Carulli, 2025; Festa & Thiele, 2011; Finney & O'Halloran, 2003; Fitisemanu & Padilla-Benavides, 2024; Ge et al., 2022; Maung et al., 2021; Robinson & Winge, 2010).

Skeletal muscle provides a powerful model for dissecting Cu biology, due to its well-defined transcriptional program and the extensive knowledge of its transcription factors, chromatin remodelers, and lineage regulators (Berkes & Tapscott, 2005; Brack & Rando, 2012; Buckingham & Rigby, 2014; Chang & Rudnicki, 2014; Cho et al., 2015; Du et al., 2012; Faralli & Dilworth, 2012; Hernandez-Hernandez et al., 2013; Joung et al., 2018; Kablar et al., 1997; Montarras et al., 2013; Nasipak et al., 2015; Ohkawa et al., 2007; Padilla-Benavides et al., 2020; Padilla-Benavides et al., 2015; Padilla-Benavides et al., 2017; Padilla-Benavides, 2022, 2023; Pallafacchina et al., 2010; Rudnicki & Jaenisch, 1995; Sambasivan & Tajbakhsh, 2015; Seale et al., 2000; Venuti et al., 1995; von Maltzahn et al., 2013; Witwicka et al., 2019; Yablonka-Reuveni et al., 1999; Yin et al., 2013). With its high metabolic demand, intrinsic requirement for Cu, and remarkable regenerative capacity, skeletal muscle is uniquely suited to uncover novel Cu-dependent mechanisms. Our group has established Cu as a key regulator of skeletal muscle differentiation and function (Carulli, 2025; McCann, 2022; Tavera-Montanez et al., 2019; Verdejo-Torres et al., 2024; Vest et al., 2018). During adult myogenesis, satellite cells progress from quiescence to proliferation, undergoing a metabolic shift from fatty acid oxidation to glycolysis and ultimately to oxidative phosphorylation, a transition that requires Cu for mitochondrial biogenesis and function (Montarras et al., 2013; Remels et al., 2010; Ryall et al., 2015). Indeed, disruption of Cu-dependent mitochondrial protein synthesis impairs both myogenesis and muscle regeneration (Hamai et al., 1997; Moyes, 2003; Moyes et al., 1998; Moyes et al., 1997; Wagatsuma & Sakuma, 2013). Beyond its metabolic role, we have shown that Cu also contributes to transcriptional and post-transcriptional regulatory processes that support myogenesis (McCann et al., 2022; McCann, 2022; Tavera-Montanez et al., 2019; Verdejo-Torres et al., 2024; Vest et al., 2018; Whitlow et al., 2023). Furthermore, our transcriptomic analyses revealed that Cu availability directly influences cellular protein synthesis capacity through specific Cu-BPs (Verdejo-Torres et al., 2024).

Through metalloproteomics analyses, we identified murine cysteine-rich intestinal protein 2 (mCrip2) as a novel Cu-BP expressed in primary myoblasts (Verdejo-Torres et al., 2024). mCrip2, a conserved member of the CRIP family, has been linked to autophagy (Chen et al., 2021), Wnt-regulated neural crest migration (Wei et al., 2011), and glycolysis regulation in cancer (Zhou et al., 2018). Biochemical assays showed high-affinity Cu binding (Chen et al., 2021; Verdejo-Torres et al., 2024), while CRISPR/Cas9-mediated deletion of *mCrip2* impaired myoblast differentiation (Verdejo-Torres et al., 2024). Confocal and CUT&RUN analyses further revealed that mCrip2 localizes to both cytosol and nucleus of proliferating and differentiating myoblasts and binds to a defined set of promoters in a Cu-dependent manner (Verdejo-Torres et al., 2024).

Remarkably, RNA-seq analyses of *mCrip2* -deficient myoblasts revealed robust downregulation of genes involved in mRNA processing, ribosome biogenesis, translation, and transcriptional/chromatin regulation (Verdejo-Torres et al., 2024). Among these categories, ribosomal genes such as *RpsA* and *Rps8* , critical for 40S assembly and rRNA processing, were consistently reduced in knockout cells, suggesting that Cu-BPs like mCrip2 integrate metal homeostasis to the translational machinery. To test this hypothesis, we examined global protein synthesis in *mCrip2* -deficient cells in comparison to control wild-type (WT) and myoblasts transduced with an empty vector treated with cycloheximide (CHX), a classical translation inhibitor (Hayashi et al., 2022; Li et al., 2020). Our previous work showed that Crip2-deficient myoblasts fail to differentiate, yet supplementation with non-toxic CuSO _4_ increased cell retention on culture plates (Verdejo-Torres et al., 2024), indicating a survival advantage despite impaired differentiation. Guided by the RNA-seq data, we next quantified protein synthesis using SDS-PAGE and Coomassie Brilliant Blue staining.

First, we titrated CHX concentrations in differentiating WT myoblasts to identify a working dose that inhibited protein synthesis by ~50% without causing widespread cell detachment ( **Fig. 1A, B** ). A concentration of 5 µg/ml was selected for subsequent comparisons as higher concentrations led to extensive cell detachment without further loss of protein content. CHX consistently reduced protein synthesis regardless of insulin or Cu supplementation, although detachment was more pronounced under basal differentiation (-Ins/-Cu) conditions. Guided by RNA-seq data showing that *mCrip2* loss downregulates genes involved in translation, we next examined the impact of *mCrip2* deletion on protein synthesis using SDS-PAGE followed by Coomassie Brilliant Blue staining. We utilized three independent primary *mCrip2* knockout myoblast lines previously reported ( **Fig. 1C** ). To directly compare global protein synthesis under different conditions, lysates were normalized by both total cell number and extraction volume rather than by protein concentration. This approach allowed us to quantify total protein content per cell, thereby capturing differences in overall protein accumulation rather than masking them through post-extraction normalization. Equal numbers of viable myoblasts were counted and lysed at a constant buffer-to-cell ratio, ensuring that variations in Coomassie-stained total protein reflect genuine changes in protein synthesis capacity rather than differences in cell survival or extraction efficiency. Gel imaging and densitometric analyses showed that under basal differentiation conditions ( **Fig. 1D** ) or with insulin supplementation ( **Fig. 1E** ), *mCrip2* loss reduced total protein content by 20-30% relative to WT or EV controls. Strikingly, Cu supplementation fully restored protein levels in knockout cells to those of WT and EV ( **Fig. 1F** ). Statistical analysis comparing *mCrip2* -deficient myoblasts with and without Cu supplementation demonstrated a significant increase in total protein levels upon Cu treatment, indicating that this transition metal restores the protein synthesis capacity compromised by *mCrip2* depletion ( **Fig. 1G** ).

Together, these findings establish that loss of *mCrip2* causes a partial translational deficit, and that Cu supplementation can rescue this defect. While the contribution of Cu-BPs to transcriptional and translational control in mammalian cells remains largely unexplored, our data identify mCrip2 as a Cu-responsive regulator that integrates metal homeostasis with protein synthesis, an essential biosynthetic process for skeletal muscle differentiation. This work expands the paradigm of Cu biology, extending its known roles in mitochondrial function and redox balance to include direct regulation of the translational machinery.

## Methods

**Cell culture**

Murine primary myoblasts were purchased from iXCells Biotechnologies (10MU-033) and cultured in growth medium containing 1:1 *v/v* DMEM:F-12 (Life Technologies), 20% fetal bovine serum (FBS, Life Technologies), 25 ng/ml of basic fibroblast growth factor (FGF; Sigma Aldrich), 5% chicken embryo extract (C3999; United States Biological Corporation) and 1% antibiotics (penicillin G/streptomycin, Gibco) in a humidified atmosphere containing 5% CO _2_ at 37°C.

Primary myoblasts were seeded at 6x10 ^4^ cells/cm ^2^ for all differentiating conditions. Specific culture conditions established for differentiating myoblasts are indicated in the figure legend. Differentiation was induced by shifting the cells into differentiation media consisting of DMEM (Life Technologies), 2% horse serum (Life Technologies), 1% antibiotics, supplemented or not with a mixture of Insulin/Transferrin/Selenium (Gibco) and 30 µM CuSO _4_ as described (Tavera-Montanez et al., 2019; Verdejo-Torres et al., 2024; Vest et al., 2018). Myoblasts were cultured on plates treated with 0.01% Matrigel (Corning, Inc.)

To produce lentiviral particles, HEK293T cells were purchased from ATCC (Manassas, VA). HEK293T cells were maintained in growth media containing DMEM supplemented with 10% FBS and 1% antibiotics in a humidified incubator at 37°C with 5% CO _2_ .

**Plasmids construction, virus production, and transduction of primary myoblasts**

We generated *mCrip2* knockout (KO) primary myoblasts using CRISPR/Cas9 technology (Verdejo-Torres et al., 2024). Guide RNAs (gRNAs) of 20 nucleotides were designed to target sequences upstream of a 5′-NGG protospacer-adjacent motif (PAM) within the intron of interest, and their specificity was confirmed by whole-genome searches to minimize off-target effects (Verdejo-Torres et al., 2024). CRISPR/Cas9 lentiviral constructs were prepared following the lentiCRISPRv2 oligo cloning protocol (Sanjana et al., 2014). Briefly, complementary sense and antisense oligos (Integrated DNA Technology, IDT) were annealed, phosphorylated, and cloned into the BsmBI sites downstream of the human U6 promoter in the lentiCRISPRv2 plasmid (a gift from Dr. F. Zhang; Addgene plasmid #52961; (Sanjana et al., 2014; Shalem et al., 2014)). An empty vector (EV) encoding Cas9 but lacking sgRNA was used as a null KO control.

To generate lentiviral particles, 5 × 10 ^6^ HEK293T cells were seeded in 10 cm dishes. The following day, cells were transfected with 15 µg of the *mCrip2* sgRNA-containing CRISPR/Cas9 vector together with packaging plasmids pLP1 (15 µg), pLP2 (6 µg), and pSVGV (3 µg), using Lipofectamine 2000 (Invitrogen) according to the manufacturer’s instructions. After 24 h, the medium was replaced with 10 ml DMEM supplemented with 10% FBS. Viral supernatants were collected at 24 and 48 h, filtered through a 0.22 µm syringe filter (Millipore), and used for myoblast transduction. Primary myoblasts (2 × 10 ^6^ ) were infected with 5 ml of filtered viral supernatant supplemented with 8 μg/ml polybrene (Sigma Aldrich), as described (Tavera-Montanez et al., 2019; Verdejo-Torres et al., 2024). After overnight incubation, cells were selected in growth medium containing 2 μg/ml puromycin (Invitrogen), and stable lines were maintained in 1 μg/ml puromycin.

**Antibodies and western blot**

Primary rabbit anti-Crip2 (A9038) and anti-GAPDH (A19056) antibodies were purchased from ABclonal. Differentiating primary myoblasts were lysed in RIPA buffer [10 mM PIPES pH 7.4, 150 mM NaCl, 2 mM EDTA, 1% Triton X-100, 0.5% sodium deoxycholate, 10% glycerol] supplemented with protease inhibitors (Thermo Fisher Scientific). Lysates were sonicated for 6 min (30 s on/off cycles, mild intensity) using a Bioruptor UCD-200 (Diagenode, NJ), and protein concentrations were determined by the Bradford assay (Bradford, 1976). Equal amounts of protein (20 µg) were resolved on 10% SDS-PAGE gels and transferred to PVDF membranes. Membranes were incubated overnight at 4°C with primary antibodies (1:1000), followed by species-specific secondary antibodies for 2 h at room temperature. Detection was performed using HRP substrate for enhanced chemiluminescence (ECL; Tanon, Abclonal Technologies). Band intensities were quantified by densitometry using ImageJ v1.8 (NIH; (Schindelin et al., 2012)).

**Inhibition of protein translation by cycloheximide**

Primary myoblasts were seeded at 6x10 ^4^ cells/cm ^2^ , allowed to reach confluency and differentiated in the presence of absence of insulin and Cu, as indicated in the figure legends, and incubated for 24 h with increasing concentrations of cycloheximide (CHX; 5-50 µg/mL). At harvest, total viable cell numbers were determined using a Cellometer. Cell pellets were then resuspended in lysis buffer at a constant ratio of buffer volume to cell number (typically 100 µL per 1x10 ^6^ cells) to ensure equivalent extraction efficiency across samples. Samples were sonicated at medium intensity for 5 min with 30 s on-off cycles in a Bioruptor. Equal volumes of lysate (10 µl), corresponding to the same number of cells, were loaded directly onto 10% SDS-PAGE gels for Coomassie staining. This approach allowed us to compare total protein content per cell across conditions, independent of differences in viability or proliferation. Densitometric analyses of total protein per lane were performed with ImageJ software v.1.8 (NIH; (Schindelin et al., 2012)).

**Statistical analysis**

In all cases, the data represents the mean of three independent biological replicates ± SE. Statistical analyses were performed using Graph Pad Prism 7.0b (Dotmatrics, Boston, MA). Multiple data point comparisons and statistical significance were determined using one-way analysis of variance (ANOVA) followed by Bonferroni multiple comparison tests. Experiments where p< 0.05 were considered statistically significant.

**DATA AVAILABILITY**

Genomic data sets have been deposited and published in the Gene Expression Omnibus (GEO) (accession no. GSE252162; (Verdejo-Torres et al., 2024)).

**DISCLOSURE AND COMPETING INTERESTS STATEMENT**

The authors declare no competing interests.
